# An Economic Geography of the United States: From Commutes to Megaregions

**DOI:** 10.1371/journal.pone.0166083

**Published:** 2016-11-30

**Authors:** Garrett Dash Nelson, Alasdair Rae

**Affiliations:** 1 Department of Geography and Society of Fellows, Dartmouth College, Hanover, New Hampshire, United States of America; 2 Department of Urban Studies and Planning, University of Sheffield, Sheffield, United Kingdom; Iowa State University, UNITED STATES

## Abstract

The emergence in the United States of large-scale “megaregions” centered on major metropolitan areas is a phenomenon often taken for granted in both scholarly studies and popular accounts of contemporary economic geography. This paper uses a data set of more than 4,000,000 commuter flows as the basis for an empirical approach to the identification of such megaregions. We compare a method which uses a visual heuristic for understanding areal aggregation to a method which uses a computational partitioning algorithm, and we reflect upon the strengths and limitations of both. We discuss how choices about input parameters and scale of analysis can lead to different results, and stress the importance of comparing computational results with “common sense” interpretations of geographic coherence. The results provide a new perspective on the functional economic geography of the United States from a megaregion perspective, and shed light on the old geographic problem of the division of space into areal units.

## Introduction

For numerous reasons, ranging from the widening scale of labor markets to the integration of capital flows, many observers have suggested that the economic geography of the United States is now best understood in terms of “megaregions.” These are assumed to be large regional areas, often cutting across state lines, that are normally centered on major metropolitan hubs and include an orbit of smaller sub-centers. To divide the country into a mosaic of such megaregions, analysts typically rely on a loosely interpretive method which takes into account physical proximity, morphological integration, and cultural similarity, in order to group major and minor cities together with rural areas into coherent regional entities; e.g. Hagler [1] or Kotkin and Schill [2].

However, the empirical problem of dividing space into discrete, bounded, internally-homogenous regions has long been a vexed problem for geographers, with attempts at providing an objective method for regionalization stretching back more than a century [3–6]. While more recent scholarly work has emphasized concepts of connectivity and relationality as theoretical lines of inquiry which lead beyond the confines of bounded space [7–10], the goal of partitioning the United States into functional megaregions shows that the old problem of regional delineation remains very much alive—and unsolved. A geography based solely on what Khanna calls “connectography” [11] fails to offer a practical framework within which to define the bounded geographic areas which continue to mark out spheres of legal jurisdiction, planning authority, transportation, and political representation.

In this paper, our contribution is to offer an empirical approach to detecting and defining megaregions which takes the insights of a relational, flowing concept of geography and puts them to use in service of delineating coherent, bounded regions. We employ a data set of more than 4,000,000 commutes as a proxy for patterns of economic interconnection, given the importance of commutes in structuring the geography of labor markets [12,13]. The volume and resolution of this data set allows us to depict the interconnected nature of these labor markets at a national scale and, we hope, allows us to make a substantive methodological contribution to the study of megaregions in the United States.

We use these flows in order to provide a rigorous and evidence-based assessment of whether “megaregions” exist and, if so, what spatial forms they take. We do so through an exploration of two different approaches: one requiring visual interpretation, and the other relying strictly on algorithmic computation. In the visual interpretation method, we show how flow mapping can be used in order to show the spatial clustering of urban 'megaregions' by employing cartographic techniques that augment a visual recognition of interrelation. In the algorithmic method, we employ network partitioning software developed at the MIT Senseable City Lab [14] in order to assess the utility and reliability of a purely statistical analysis in determining the geographical break points between communities. Such a method hints at a possibility long promised by spatial scientists: a regionalization scheme which relies entirely on spatial laws, rather than contestable human interpretation. However, we caution against the idea that regional units can be incontrovertibly determined by raw mathematical analysis alone, and show how “big data” methods are dependent not only on the reliability of input sources, but also on choices about parameters, and “common sense” checks on results.

## Background

The impossibility of absolutely defining stable territorial regions has long been recognized by geographers and those working in allied disciplines. For example, in his influential 1939 essay “The Nature of Geography,” Richard Hartshorne warned that “the face of the earth is the very antithesis of a mosaic” [15]. Hartshorne pointed to the complex and highly heterogeneous distribution of features which characterizes human geography, concluding that “man does not, consciously or unconsciously, organize a region as a unit” [15]. Though this old debate has now been superseded by approaches to human geography which emphasize “relationality,” the problem of defining regional units nonetheless remains a vexing and unsolved problem. For numerous practical reasons it is necessary to stabilize space into discrete entities with well-defined edges. To list just a few examples, electoral districts, regional transit administrative zones, and municipal jurisdictions all require an evaluation of what territorial space is appropriate to treat as a “single” region from a policy and planning perspective. However borderless and intricately intermeshed the actual pattern of human geography may be, the question of how to extract functional regions and edges from the fuzziness of that pattern remains an important task for applied geographers and their counterparts in public administration.

A contemporary approach to solving this problem can be found in literature on megaregions [16,17]. The concept of the “megaregion”—like similar precedent concepts such as the “conurbation” [18] or “megalopolis” [19]—has developed in response to new patterns of economic integration operating across wider geographic scales. As the functional patterns of human geography have transformed and rendered older territories inadequate or obsolete for the purpose of managing public services, one of the key concerns in studies of these kinds has been the practical question of how to divide space into new administrative regions whose geographic logic follows the underlying functional structure of these new patterns [20,21].

“Megaregions” may well pass a common-sense test, based on a recognition of the ways in which larger areas have been substantively tied together by the forces of urban development, telecommunications, the frictionless circulation of capital, and the consolidation of both public and private institutions. However, the problem of how to empirically determine the existence and precise shape of these areas is an extension of the old geographic problem of dividing space into unit areas. In this paper, we attempt to show the functional existence of megaregions in the United States by turning to a large data set of commuting patterns and showing how “natural” patterns of community clustering can be demonstrated both through visual interpretation as well as through statistical analysis.

We believe that our results offer a new, more empirically rigorous evaluation of megaregions and demonstrate the utility of this approach in gaining a better understanding of the functional economic geography of the United States at a macro-spatial scale.

## Data and Methods

We focus on journeys to work (commutes) owing to their importance in the functioning of local and regional labor markets, and because the volume of data available provides a robust test case for the community partitioning algorithm we describe below. In section 4 of the paper, we report our results based on an analysis of more than 70,000 spatial nodes and more than 4,000,000 connections between them, using a combination of desktop and cloud computing.

Given the geography of travel to work in the United States, the county scale of aggregation was deemed too coarse and too variable to be of use. The most populous county, Los Angeles County, is home to more than 10,000,000 people while the least populous, Loving County, Texas, is home to fewer than 100. The mean county population at the time of the 2010 US Census was almost 100,000. Further, many of the nation’s more than 3,000 counties are far larger or far smaller than the metropolitan areas within which they are located, making any analysis of journeys to work at this scale problematic, in the sense that the geographic scale of analysis would be too large to identify the underlying phenomenon of journey to work patterns we seek to investigate.

Therefore, we decided to focus our analysis at a finer geographic resolution in order to capture, as far as possible, the volume and diversity of flows. For this purpose, the Census Tract was deemed the most appropriate spatial unit, since it has an average population of just over 4,000 and Census Tracts are geographically large enough to contain major numbers of employees, unlike smaller Census units such as Blocks or Block Groups. Data on journeys to work are reported for just over 74,000 Census Tracts in the United States and are available from two different sources: the American Community Survey (ACS) commuting and workplace data and the Longitudinal Employer-Household Dynamics Origin-Destination Employment Statistics (LODES). These complementary datasets can help us answer important questions about the economic, demographic and spatial relationships associated with daily journeys to work for more than 130 million Americans but they differ in several important respects [22], as we explain below.

As the name suggests, the American Community Survey commuting and workplace data are derived from a nationwide survey of employees, asking where they worked “last week.” The ACS is a large, continuous survey of around 3,500,000 addresses per year and data are available in one, three or five year estimate periods. The latter is based on 60 months of data and reflects the characteristics of an area over the entire period. By contrast, in the LODES dataset, employment location is reported by employers, and, if the employer has more than one location, this may not be the same location where an employee actually works [22]. LODES job counts are released down to the Census Block Level, whereas ACS commuting and workplace data are available down to Census Tract level.

Our considered view is that LODES data may be most appropriate for the analysis of commute patterns at more local levels (e.g. within a single metropolitan area) rather than at the national scale, as we report in this paper. In short, each dataset is useful in its own right and could be usefully deployed to carry out the analysis reported here. However, we have used the ACS 5-year estimates in order to provide the most robust measure of nationwide commute patterns, in the absence of the now-discarded travel to work questions from the Long Form Census.

### The ACS 2006–2010 Census Tract Flow Dataset

**Fig 1:**
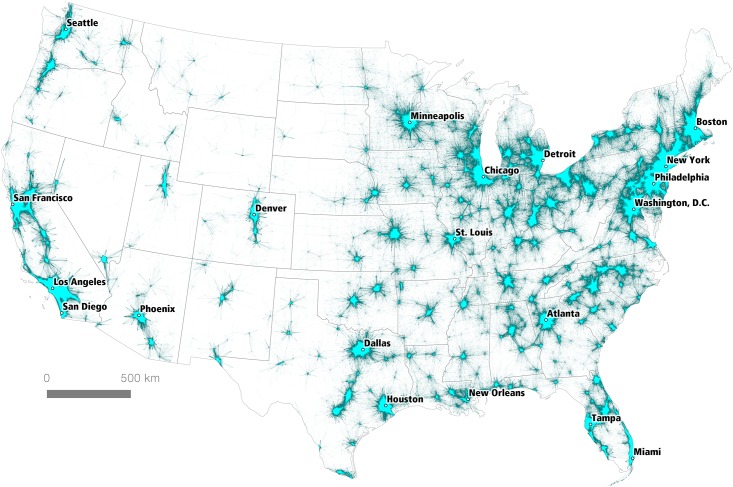
Tract-to-Tract Commutes of 160km or less.

The dataset we used contains origin and destination information for 4,156,426 journey to work flows between 74,002 Census Tract centroids in the United States and Puerto Rico. We computed the Euclidean distance between origin and destination tract centroids, in order to provide a distance estimate between workplace and residence. Additional fields were added for tract, county and state FIPS codes in order to allow for subsequent filtering of the data using these geographies. Within the United States (minus Puerto Rico), this left a total of 4,155,548 tract-to-tract flows associated with 130,163,663 total commuters. When filtered by distance (d < 160km) and plotted on a map of the Lower 48 states, this produces a geographic representation of the urban fabric of the United States that shows how individual areas are connected, as seen in Fig 1. A distance of 160km (c. 100 miles) was chosen because it includes all but the most extreme commutes, and accounts for 97.4% of all point-to-point commuter flows in the data set. Fig 1 illustrates the interconnectedness of some larger geographic areas, such as the northeastern seaboard, which evokes Gottmann’s original “megalopolis,” spanning the area from Boston in the north to Washington, D.C. in the south. This representation requires further scrutiny, and is therefore the subject of the remainder of the paper.

### A visual heuristic approach to regional delineation

When attempting to make sense of large and complex spatial datasets, it is possible to take a number of different methodological approaches. Here we examine two of the most common: a visual heuristic approach and an algorithmic approach. According to Smelcer and Carmel [23], the cognitive work we do with maps is simplified using visual heuristics, and this is certainly the case when we compare a large commuter data table with millions of cells to the kind of cartographic representation shown in Fig 1. However, we do not concur with Smelcer and Carmel when they state that “visual heuristics differ from algorithms, which are *guaranteed to provide a correct solution”*‘ (emphasis added; cf. Anderson, [24]). As we shall see below, the extent to which a “correct solution” can be derived depends upon user-defined parameters, the nature of the algorithm used and the underlying epistemological position of the researcher. Nonetheless, we do believe that the algorithmic approach we develop confers many advantages above and beyond the visual heuristic and has significant potential in adding value to the visual approach, particularly with respect to discriminating the dividing lines or overlaps between closely-integrated regions.

A heuristic approach is, put simply, one which involves exploration through trial and error to produce results which are useful, but not necessarily optimal. Simon [25] refers to this as the concept of “heuristic search” in which sub-optimal but effective “satisficing” solutions are arrived at. We take this visual approach with the ACS commuting and workplace data by working iteratively towards representations which identify core labor market areas based on networks of flows. In addition to providing a visual ‘sense check’ on the underlying data, this also provides a useful reference point for the algorithmic approach described later.

In order to operationalize the visual heuristic approach, we employed Rae’s “principles for the orderly loss of information” in which *iterative loss* and *optimal compromise* are key tenets [26]. In the former, different parameters are applied in order to filter out the “noise” in a dataset; akin to Boulding’s maxim that “knowledge is always gained by the orderly loss of information” [27]. The latter principle—optimal compromise—is an explicit recognition that even in empirically-driven studies the results are never perfect, which again echoes Simon’s ‘satisficing’ principle. These also prove a useful way to operationalize Polya’s mathematical problem solving approach in which the visual approach to understanding is given a prominent role [28].

As we report in the next section of the paper, the visual heuristic approach in practice involved filtering the ACS data based on Euclidean distance and flow volume thresholds in order to produce a labor market regionalization of the United States which was both empirically driven and visually effective. Ideally, such an approach would also include network distance and travel time parameters, but these introduce a level of computational complexity beyond that which can be accommodated here.

Fundamentally, we are seeking to test the efficacy of the visual heuristic approach *vis-a-vis* the algorithmic approach to regional delineation. We acknowledge that empirical evidence suggests a commuting tolerance of 30 to 45 minutes for most workers [29] but since we are focused on the identification of larger economic regions, our visual heuristic approach uses the 50 miles figure (80.5km) for “long distance commutes” cited by Rapino and Fields in their analysis of mega commuting in the United States [30]. We use this longer distance in an attempt to capture the vast majority of commuters within metropolitan areas.

### An algorithmic approach to regional delineation

Newman identifies the problem of detecting and describing community structures within network data as “one of the outstanding issues in the study of networked systems” [31]. A “community” in a networked data set refers to a set of nodes in which the density of relations is stronger internally within the community than it is externally with members of different communities, or, as Newman describes it, “the appearance of densely connected groups of vertices, with only sparser connections between groups” [31]. In a visual representation, network data of this type is often rendered as dots (vertices or nodes) connected by lines (variously called connections, edges, or arcs), and communities are shown as dots with many lines connecting between one another, often circled by a provisional border indicating the limits of the community. Although this usually this means that the nodes in a community are visually represented as spatially proximate, the mathematical definition of ‘community’ in this sense does *not* rely on the spatial positioning of the nodes, only on the density of connections. Community structure defined in this way is topological, not topographical.

The validity and strength of a proposed community structure can be measured by the variable *modularity*, which Newman and Girvan define as a function of the total number of connections within a network set which lie entirely *within* a proposed community boundary (as opposed to *across* community boundaries) [32]. As Newman and Girvan note, however, this cannot be evaluated as a simple fractional measure, since assigning *all* of the members in a full data set to just one single community would therefore result in a perfect score. Their proposed modularity variable therefore tests intra-community connections against a perfectly random assignment of communities. A value of 0 indicates a partitioning of nodes into communities which is “no better than random,” while a value of 1 indicates “networks with strong community structure” [32].

By treating the ACS commuter data set as a network consisting of nodes (census tracts) and edges (commutes), we can apply the algorithmic techniques which statisticians and data scientists have developed for community detection and test whether it is possible to detect the signature of “natural” community groupings in the pattern of commuter geography. Several software packages exist which employ various algorithmic approaches to community detection; we chose to use the ‘Combo’ package developed by Sobolevsky et al. at MIT’s Senseable City Lab [14].

Combo achieves high-accuracy partitioning, measured by the modularity score of the output communities, at the price of a relatively more intense computational task. Sobolevsky et al. report good results for Combo on a desktop computer with data sets of up to 30,000 nodes; our set contains slightly over 70,000 nodes, and consequently required the use of high-performance computing hardware. We therefore compiled Combo from source on an Amazon Web Services EC2 (memory-optimized) virtual machine with 122 GB of RAM running Ubuntu Linux.

Combo requires its input data in the Pajek network format. This format requires nodes to be identified by sequential natural numbers. We wrote a Python script to iterate through the database of commutes, assigning identifiers to each census tract node with at least one commute origin or destination as the “vertices” of the network, and using these identifiers, together with the flow volume, to list the “arcs” of the network. We then used this Pajek file as an input source for the Combo software. Computing time for different versions of the data set ranged from 8 to 12 hours. After each compute, we merged the output, in which Combo returns community identifiers for each input node, back into a spatial file with coordinate data for each census tract. This gave us mappable data of census tracts and their communities as assigned by the community detection software.

Again, such an approach means leaving out the spatial data attached to each node during the computational phase. Unlike, for example, the partitioning algorithm developed by Gaudart et al., which analyzes community clustering based on nodes’ proximity in space [33], network analysis in Combo considers *only* the strength of a connection between two nodes, not their physical nearness to one another. In our case, the ‘strength’ of a connection is determined by the volume of the commuter flow between two census tracts.

The geography-blindness of the algorithm therefore also allows us to provide a test case for Tobler’s “First Law” of geography: the premise that ‘near things are more related than distant things’ [34]. Census tracts near to each other should, according to this logic, have stronger commuter connections to one another than census tracts far apart. Consequently, even though the partitioning algorithm is not considering nodes’ locations in space, it *should* produce communities which are spatially clustered, if the structure of commuter patterns obeys the expected rule of more connections between spatially-proximate nodes, and fewer connections between spatially-distant nodes.

## Results

In this section of the paper we present the results of our visual and algorithmic analyses. In the first part, we look at the extent to which a visual approach to understanding the ACS commuting and workplace data can help us identify natural “communities” or “regions” of interaction within which journeys to work take place. These provide a visual approximation of labor market areas of the kind discussed in the academic literature for decades [20, 35–38]. Rather than attempt to do this at the scale of the whole United States, we focus our visual heuristic on California and the Minneapolis-St. Paul metro area. The former provides a good example of an apparently polycentric urban network, with multiple centers and a more complex commuting structure, as described previously by Cervero and Wu [39,40]. The latter provides a good example of an apparently monocentric urban commute structure, dominated by a core employment zone in the center, of the kind studied in the past by, inter alia, Bogart and Ferry [41] and Richardson [42]. Both approaches can help us understand more about the underlying economic geography of major metropolitan areas of the United States.

The second part of this section is comprised of the results of our algorithmic approach to assigning census tracts into ‘natural’ communities based on their relational position within the network data set. We show the promising results of such an algorithmic evaluation and the success of the Combo software package in making fine-scale discrimination between the edges of megaregions. We also show some of the difficulties and data outliers produced by such an approach, and suggest some qualifications which must be attached to purely statistical analyses.

### Identifying polycentric and monocentric spatial structures

**Fig 2:**
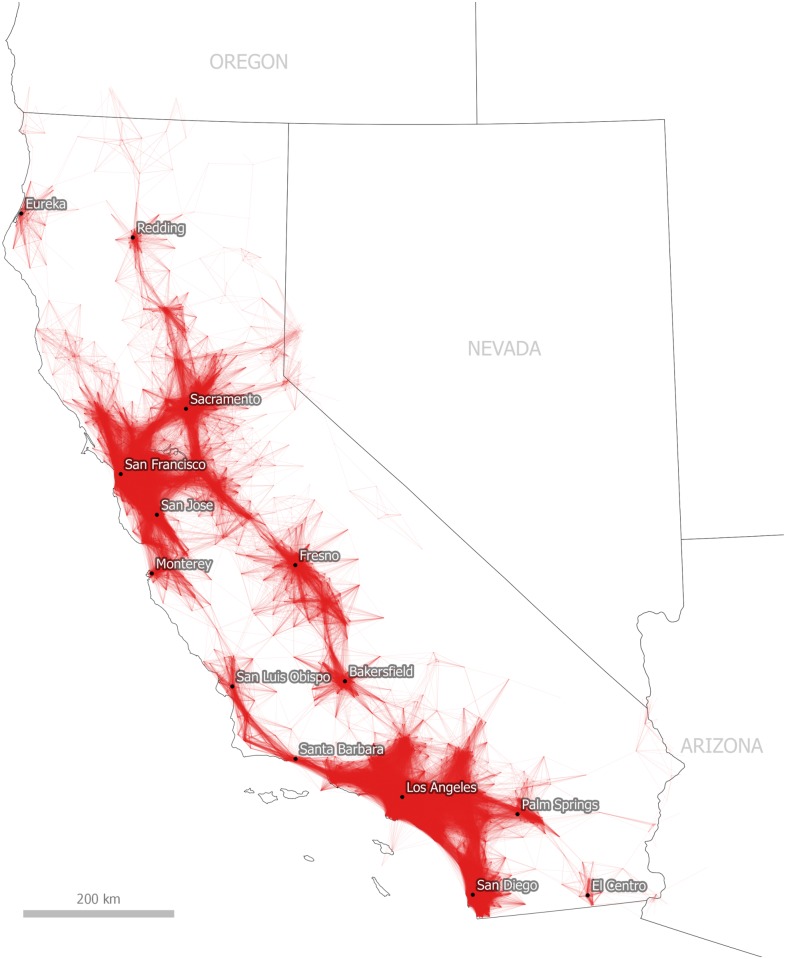
Tract-to-Tract Commutes of 80km/50 miles or less in California.

The simple question we first hope to answer by taking a visual heuristic approach to commute data is whether it is possible to divide geographic space by taking an iterative approach to filtering and visualizing the ACS commuting and workplace dataset. Fig 2 represents a first step towards this objective. We present all journey to work flows of 50 miles (80.5 km) or less which begin or end in California. Immediately, we can see what appear to be a number of separate functional economic zones. For example, we can see a large interconnected urban region spanning from San Luis Obispo on the California coast, extending through Los Angeles and San Diego in the south and Palm Springs in the east. We can also observe another large commuter region which takes in the metropolitan areas of San Francisco, Sacramento, San Jose and Monterey. Additionally, we observe smaller, separate interconnected commuter regions including Eureka and Redding in the North, Fresno and Bakersfield in the Central Valley and El Centro in the south. These patterns do, of course, map onto underlying patterns of population density but they also provide valuable additional information in relation to the connectivity of these areas with each other.

**Fig 3:**
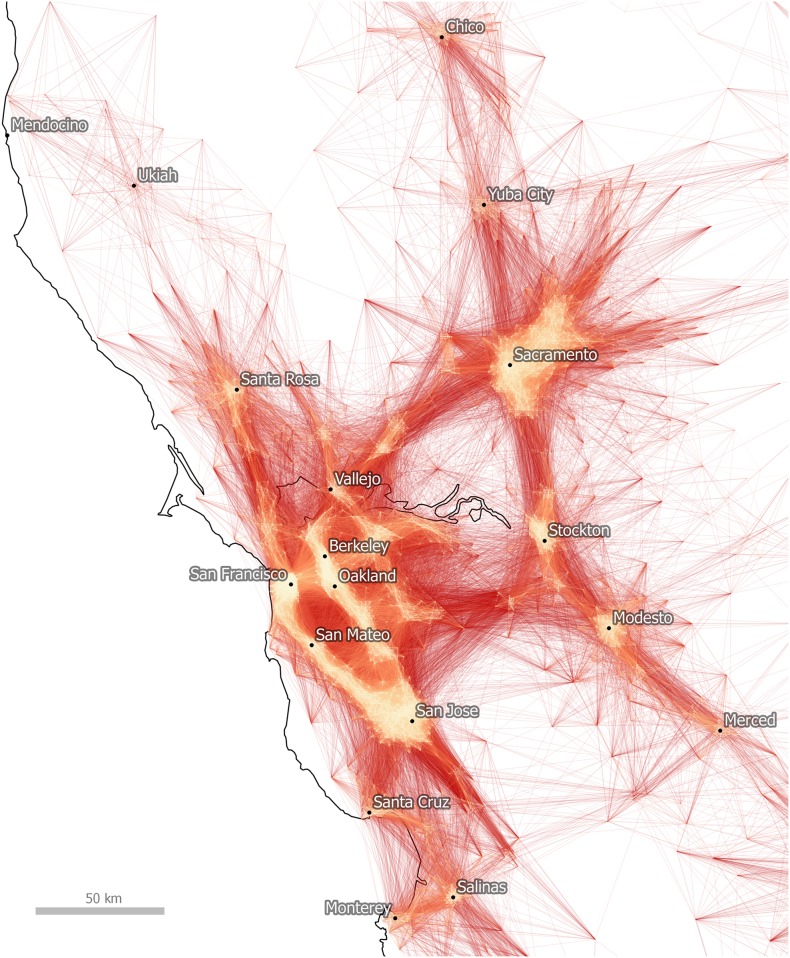
Tract-to-Tract Commutes of 80km/50 miles or less in the Bay Area.

This initial plotting of commutes is quite useful in that it provides a simple visual depiction of economic linkages and we can begin to understand the spatial structure of commuting in California. If we refine this representation still further, as in Fig 3 where we focus on the San Francisco Bay Area, a more detailed representation of a polycentric urban region emerges. In this representation we display longer, lower volume commutes in darker shades of red and shorter journeys in lighter shades of orange, in order to help the viewer identify the main employment centers. In Fig 3 they include San Francisco, Oakland and Sacramento, but also Stockton, Modesto and Santa Rosa. Nonetheless, it is difficult to determine from this view the extent to which these links are statistically significant and whether this nexus of economic activity constitutes a single functional zone in and of itself. We deal with this issue in the next section of the paper through the algorithmic method.

**Fig 4:**
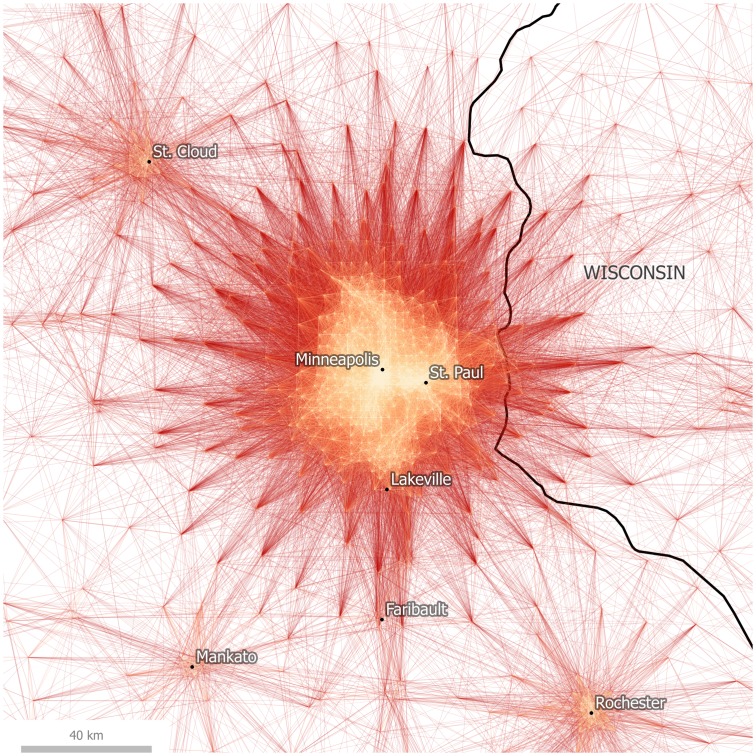
Tract-to-Tract Commutes of 80km/50 miles or less in Minneapolis-St. Paul.

Before doing so, we present the case of Minneapolis-St. Paul, which represents a rather different case to that of California and the Bay Area. In Fig 4 we have plotted tract to tract commutes centered on Minneapolis-St. Paul, which appears to form a major monocentric employment zone in Minnesota, also extending into western Wisconsin. More distant satellite cities such as St. Cloud to the north west and Rochester to the south east are less strongly connected to this dominant urban employment destination, but it is difficult to know the extent to which they are functionally separate from a visual inspection alone, and this is the point: cognitively, the viewer makes assumptions about the modularity of a network based on visual representations like the commute maps shown here, but this is imprecise and somewhat subjective. The mapping of flows alone can only take us so far if we are interested in knowing more about the underlying network structure of the data.

Having said this, a visual approach provides a very useful method in and of itself because it allows us to interpret data spatially, quickly identify anomalous connections and possible sources of error (cf. Anselin [43]). The next section of the paper now focuses on algorithmic community partitioning and allows us to gauge whether the processes of visual cognition (which we can think of here as a ‘manual modularity’ approach) initiated by the flow maps above are reflected in the results of the algorithmic approach. It would appear that we *can* identify natural economic communities or regions from a visual inspection alone, but for real-world applications such as regional transit planning, where statistical accuracy is required, this is not sufficient.

### Algorithmic community partitioning

In their algorithmic partitioning of data from telephone calls, Ratti et al. [44] and Sobolevsky et al. [45] found promising results which exhibited a high degree of geographic contiguity. Kallus et al. report similar results using social media interactions [46]. We sought to test whether similarly robust conclusions could be drawn from applying these same partitioning algorithms onto commutes which, unlike telephone calls and social media interactions, are far more closely bound to the physical structure of existing places.

In a trial run of the ACS data set limited to commutes both originating and concluding within the state of Massachusetts, Nelson produced a Combo-generated partition of that state into nine communities [47]. These nine communities were all geographically contiguous, and, moreover, matched closely with both lay interpretations and existing administrative divisions of that state’s regions. This initial test lent credence to the theory that commuter patterns would exhibit an algorithmically-legible grouping into “natural” communities centered on major economic/employment hubs.

In our first run of the national data, Combo produced a partitioning with a modularity score of > 0.9, and we found a large number of strong communities centered on major cities. However, the initial output communities also exhibited a considerable amount of “noise” when evaluated visually. Certain census tracts were assigned into communities that displayed little or no geographic sensibility and were confusingly scattered across the entire United States.

We proceeded through several steps to achieve a more accurate partitioning—“accuracy,” in this case, determined according to an interpretive standard of where geographic clusterings “should” be. First, we corrected a data error wherein the FIPS codes for tracts were being mishandled due to the loss of leading zeroes. Second, we stripped all commutes with origins or destinations in Alaska, Hawaii, and Puerto Rico, under the logic that these areas are not functionally integrated into the mainland United States through commuter behaviors. Third, we stripped the data of all “same-origin” commutes; that is, commutes whose origins and destinations lie in the same census tract. Fourth, we stripped the data of “orphan” nodes, that is, census tracts which are not the origin or destination of any commutes in the ACS data set. Fifth, we experimented with different tolerances of maximum commute length, in order to eliminate “ultra-commutes,” like those which stretch across the entire continent, from the data set. Such commutes may reveal significant economic ties between places (such as New York and Los Angeles) but for the purposes of identifying geographically coherent megaregions they must be excluded. Sixth, we experimented with different ways of assigning connection weight based on the Census’s variables of commute volume and margin of error. Seventh, we experimented with limiting Combo to a maximum number of total output communities.

Each time we iteratively modified these input parameters, we compared the output results with the visual heuristic method of regionalization. Our goal was to minimize the number of output regions which exhibited spatial incoherence. In general, Combo produces the most successful partitioning in areas where nodes are well-linked in the data set to many other nodes, such as is found in major metropolitan regions. Difficulties arise in nodes which are weakly connected to each other or which, due to small populations, have only a small number of commuters traveling to locations which are weakly centralized on major employment hubs. Because these weakly-linked nodes could be assigned into almost any different community with little result on the achieved modularity score, these nodes often caused trouble with geographic coherence, as the algorithm assigned them to far-flung communities which made little sense from an interpretive standpoint. By iterating through various stages of parameterization with the input data, we sought to minimize the scattering effect of these weakly-linked nodes. For a more detailed explanation of the impact of parameter modification on network modularity, Sobolevsky et al. provides a useful additional point of reference [14]. In general, however, reducing the distance parameter to a level which matched realistic commute distances, the results were improved.

**Fig 5:**
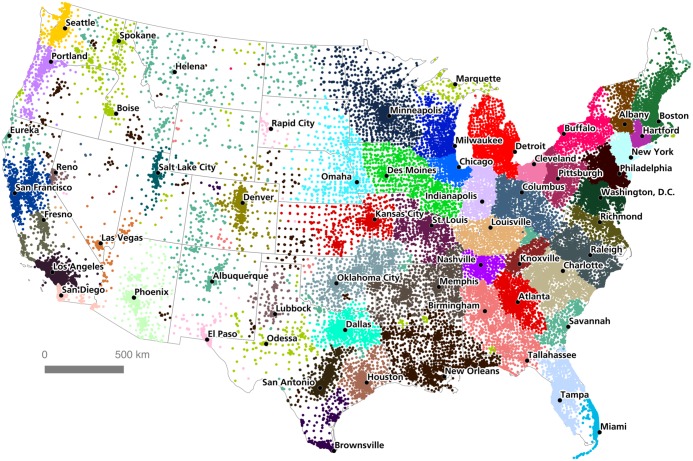
Community partitioning for the Lower 48 States, by tract.

After several iterations, we found that the most successful national-level partitioning was produced by a data set which stripped all commutes with a Euclidean distance ≥ 262 kilometers, assigned a connection weight *w* where *w* = (estimated commutes)/(margin of error), and limited Combo to 50 output communities. The modularity score of this partitioning was 0.948469: extremely high according to the expected variation of such a reported by Newman and Girvan [32]. Fig 5 shows the results of this computation, with census tracts in the contiguous United States color-coded according to their assigned community.

As is evident in this visualization, Combo was able to divide the contiguous United States into geographically-contiguous regions which are interpretively recognizable as 'megaregions' with major cities at their centers: for example, Greater Chicago (blue in Fig 5), Washington D.C-Baltimore (forest green), Greater Miami (sky blue), Dallas-Fort Worth (teal), or Seattle (goldenrod). This offers strong evidence that commuter patterns really do divide functionally in space according to the clustering of regional labor markets, and that the structure of 'megaregions' can be detected algorithmically.

Importantly, several of these algorithmically-detected megaregions also show spatial divisions which are not immediately evident in visual interpretation. Consider, for example, the detected community in the state of Connecticut (purple). Southwestern Connecticut is strongly linked to the New York City commuter region, and most visual heuristic regionalizations would merge this area into Greater New York. Yet Combo assigned an almost perfect break at the New York-Connecticut state border, creating a discrete Connecticut region which encompasses the state of Connecticut together with the Connecticut River Valley corridor running through western Massachusetts, from the city of Springfield to the Vermont border. Again, since Combo does not know “where” these nodes are in space, and does not know which state each node belongs to, the emergence of a community border which almost perfectly follows the real jurisdictional border between Connecticut and New York is highly suggestive, indicating perhaps that commuting decisions are being modified by factors that have to do with crossing this state border. To be clear, there are still many commutes crossing between Connecticut and New York; what the algorithm finds, however, is that there is a stronger internal than external matrix of connections on either side of this edge. A similar pattern is evident along the Delaware River between New Jersey and Pennsylvania, where the New York City region breaks almost perfectly into the Philadelphia region.

There are many similar interesting conclusions from this algorithmic partitioning scheme—conclusions which would *not* necessarily be legible from an interpretive visual heuristic method. Just a few other examples include: the merger of most of Iowa together with a corridor stretching through western Illinois to Springfield (grass green); the absorption of Toledo into the Michigan community (red); the merger of the Columbus and Cincinnati metropolitan areas (slate gray) the merger of Florida’s panhandle into the Alabama commuting region (salmon); the merging of the Little Rock and Memphis commuter areas (clay brown); and the absorption of Sacramento into the California Bay Area (navy blue).

**Fig 6:**
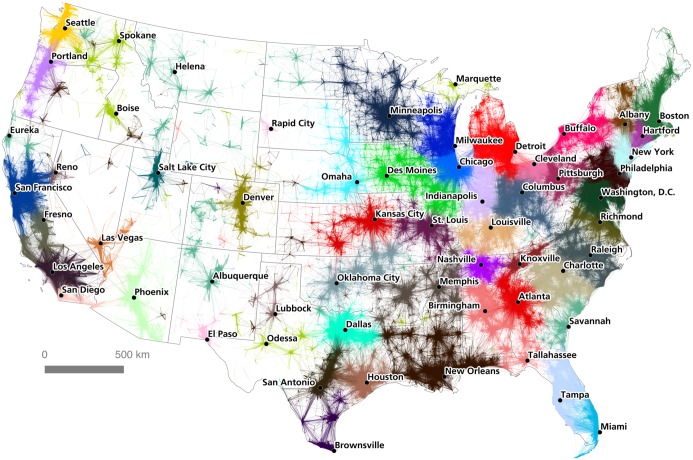
A commuter flow-based regionalization of the United States.

Instead of mapping only nodes coded according to their assigned community, we can get a better sense of the complexity of the community assignments by mapping *connections*. However, this raises the question of whether commutes should be classified according to the community assignment of their origin node or their destination node. Although the majority of commutes occur within an assigned community, some commutes stretch from one assigned community to another. Thus commutes may have one community assignment, if their origin and destination points lie within the same algorithmically-assigned community, or two assignments, if they cross between communities. On the assumption that community structure is stronger at a central point, which in commuting terms is the job end of the route, we color-coded flows according to the assigned community of their destination node. Fig 6 shows this at the national scale.

**Fig 7:**
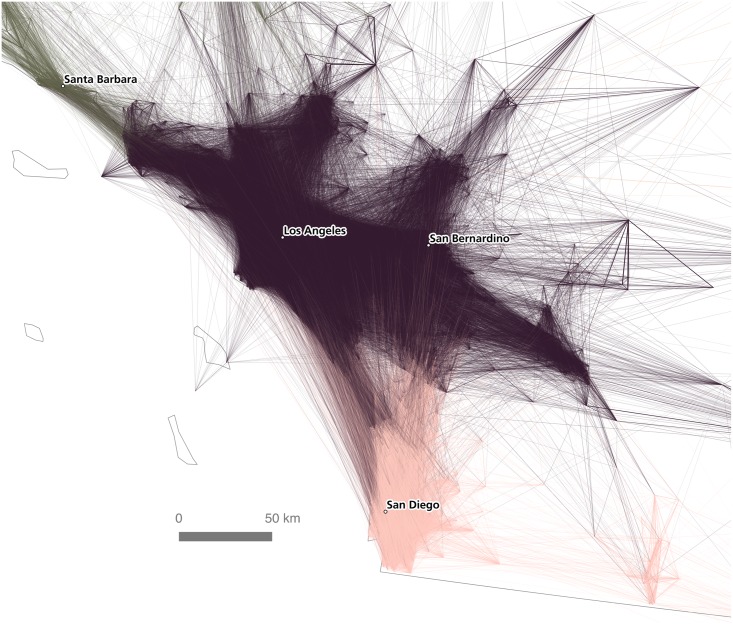
Relative density of connections between the Los Angeles and San Diego regions.

**Fig 8:**
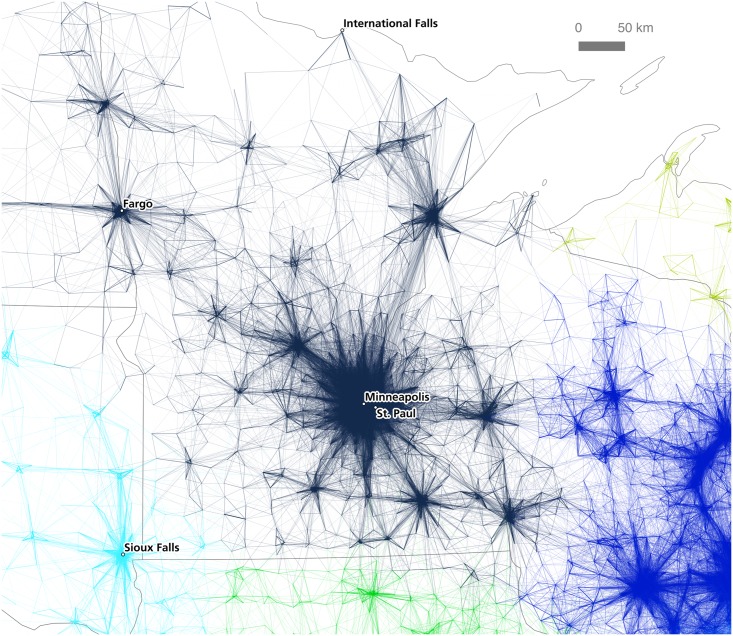
The Twin Cities region.

By coloring connections according to the assigned community of their destination node, we can see cases where neighboring communities are strongly interlinked, and also cases where communities are fairly autarchic in terms of their commuting patterns. Fig 7 shows the relative density of interconnections between the Los Angeles and San Diego detected regions (inter-community connections to mid-coast California and Las Vegas are also evident). This can be compared to Fig 8, which shows how the detected community in the Minneapolis-St. Paul region is more self-contained in terms of its commuter flows, with relatively few commutes stretching to or from neighboring communities.

**Fig 9:**
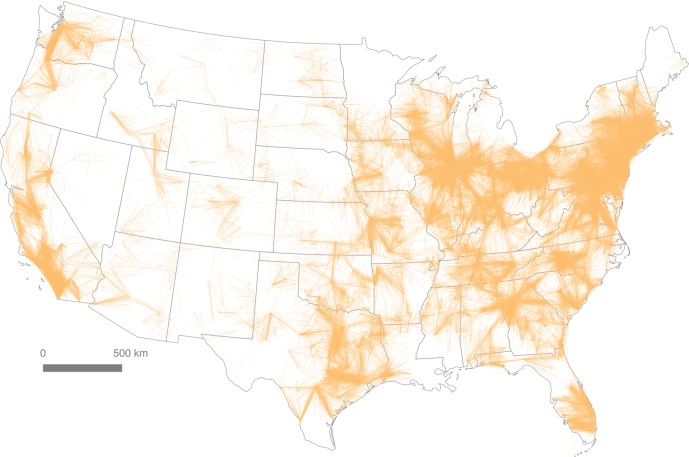
Inter-community flows for the United States.

The interweaving colors evident in such maps show just how difficult it is to discover a perfect natural break within the pattern of commuter geography. The high modularity score of Combo’s output shows that the algorithm has produced a partitioning scheme in which the vast majority of commutes are contained within a single community. However, this still leaves thousands of commutes which cross communities. Fig 9 shows every commute in the Lower 48 states where the assigned community of the origin and the assigned community of the destination are different. This gives a sense of just how incorrect it is to call these partitioned communities truly ‘independent’ or autarchic in terms of their economic geography. For instance, the northeastern seaboard, the Great Lakes, and California are heavily interlinked by commutes which stretch across regions. A large number of east-to-west flows connect between the Miami and Central Florida regions. By contrast, not a single commute to New Orleans originates from outside of the Combo-assigned New Orleans community; the Twin Cities, similarly, pulls relatively few commuters from outside its own assigned region.

**Fig 10:**
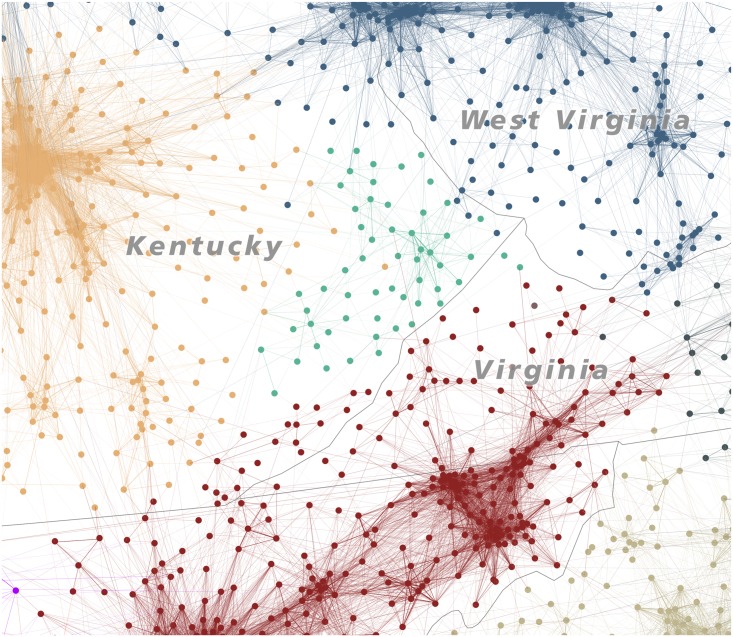
The Eastern Kentucky isolate region.

Although this algorithmic partitioning of the contiguous United States is highly satisfactory, it is not perfect. Several cases remain where nodes have been assigned to confusing, non-contiguous communities, in some cases stretching haphazardly across the entire United States. Such artifacts are especially common in less-dense areas of the country where the network structure of commutes is far weaker than in urban megaregions, and nodes are consequently less well-integrated into functional clusters. Consider, for example, the community which Combo has assigned in eastern Kentucky (jungle green). An examination of the structure of the network data (Fig 10) shows that this area really is coherent and independent in a certain sense, for it has very weak commuter relations with neighboring communities, and a reasonably strong internal structure of commuter relations. However, does it deserve its “own” unitary region? The algorithm believes that it does, whereas an interpretive method might well have included this area together with rest of Kentucky (pale brown) or the Columbus-Cincinnati-West Virginia region (gray-blue).

**Fig 11:**
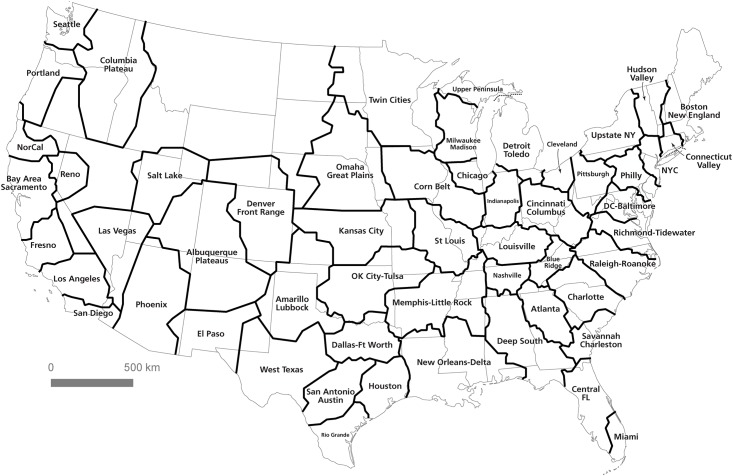
Computed communities subject to interpretive analysis.

In such cases, it becomes clear that the dream of a regionalization based purely on statistical analysis is unviable; any division of space into unit areas will have to take into account a “common sense” interpretation of the validity and cohesion of the regions resulting from an algorithmic approach. For this reason, the visual heuristic method coupled with the algorithmic method offers a good combination of human interpretation and statistical precision. The algorithm is able to detect subtle boundary definitions and evaluate edges where the human eye would struggle to draw a clear line. However, the visual method has an advantage in matching coherent regions to an interpretive understanding of regions conjoined by cultural, political, or other similarities which are not captured in the data structure of the commute patterns. Fig 11 shows the result of a combined computational-visual approach. To produce this map of U.S. megaregions, we began by tracing convex hulls around communities as assigned by the partitioning algorithm. We then overlaid these shapes onto the flow map and interpretively cleaned up boundary lines, eliminating outliers and emphasizing geographic contiguity. In some places, like the High Plains, the relatively limited level of commuter activity meant that coherent communities could not be constructed. However, the result offers what we consider to be a compelling new regionalization of the United States: one which is grounded in empirical analysis but clarified using interpretive cartographic methods.

## Reflecting upon Visual and Algorithmic Approaches

As the results of this paper strongly suggest, the geography of labor and commuter interaction really does exhibit an identifiable, coherent spatial structure. Of course, such a conclusion is not surprising, but the methods used here apply a new approach to the age-old empirical problem of regionalization. This conclusion is evident both in a visual interpretation of commutes as well as in a geography-blind algorithmic analysis of community structure within the commuter data set. In particular, the algorithmic approach provides a new, detailed view of the economic geography of the United States: one which both confirms the assumed existence of “megaregions” and lends precision to the spatial delineations between such regions. We therefore believe that empirical analysis of large spatial data sets can be used to determine, at least provisionally, how the patterns of human geography can be broken up into discrete units for practical purposes. Such an analysis is of potential use in a wide variety of fields, including transit planning, real estate, retail analysis, and infrastructure development, to name just a few. However, before concluding, it is important to discuss some of the differences between the visual and algorithmic approaches, and in particular the added value of the latter. This can be done by comparing Figs 4 and 8, which focus on the Minneapolis-St. Paul metropolitan area.

Maps and other forms of spatial data visualizations are of course not value neutral, nor are they objective; and they work cognitively in complex ways. For example, in relation to visual cognition, Peterson identified ‘pattern recognition’ as one part of the cognitive process between geographic information and knowledge output [48]. For MacEachren, one important element of pattern recognition–relevant here–is ‘grouping’, whereby regional proximity acts as a cognitive stimulus which then helps the human brain make sense of geographic data [49]. In the case of the Minneapolis-St. Paul metropolitan area, we can see how such pattern recognition and grouping might lead to different conclusions about the nature, and spatial configuration, of the wider region. For example, in Fig 4 a visual heuristic approach to the interpretation of lines and connections in the region is suggestive of a large, monocentric urban region with Minneapolis-St. Paul the dominant center.

However, Fig 8 suggests a different kind of spatial connectivity within a wider polycentric megaregion, of which Minneapolis-St. Paul is the largest part. Thus, the algorithmic approach used to develop the results presented in Fig 8 offers an important alternate view of the nature of economic connectivity across a wide area in the northern United States. Neither of these is wholly ‘correct’, but the latter does represent a computationally robust counterbalance to a potentially misleading visual-cognition approach to understanding spatial units. This brief example provides a vignette of the kinds of issues encountered in spatial economic analyses–that of taken-for-granted views of ‘natural’ spaces versus empirically derived ones. However, we recognize also that this research represents more of a departure point than a conclusive once-and-for-all regionalization of the United States. Before concluding, then, we reflect upon three further issues which arise from the foregoing analysis.

First, we need to contextualize the results in relation to what is being measured. Put simply, in this paper we have examined a form of economic activity (commuting) that takes place over a variety of geographies depending upon underlying variables such as topography, available transport modes, local and regional employment patterns and the relative health of the labor market during the time which the data were collected. This latter point is important, given that the time period of the ACS data (2006–2010) coincides with the Great Recession that emerged in the wake of the 2008 Global Financial Crisis. When more recent ACS journey to work data are released, it will open up the possibility of examining the extent to which the spatial patterns we report here are typical or not. Moreover, time-series data could open the possibility of exploring how regions consolidate, merge, or divide as economic patterns change. Beyond commuting, we also recognize that other important interactions, such as lending and credit flows or telephone calls, would most likely provide a different partitioning (e.g. Cronon [50]; Ratti et al [44])

Second, we have sought to highlight the issue of “boundedness”—or lack of it—relation to the presentation of our results. The dense mesh of overlapping flows, including many which stitch between one community and another, give credence to Hartshorne’s claim that human geography is “the antithesis of a mosaic” [15]. At the same time, however, it is clear that these flows exhibit patterns of internal consolidation within regions—patterns which are legible not only visually, but algorithmically as well. Our view is that the spatial economic fabric of the United States is a mosaic but, using another metaphor, it also appears to be a set of overlapping, interconnected cogs which, working together, constitute the functional economy of the nation. The kinds of overlaps between areas we see in Figs 7 and 9 are crucial to the functioning of any economy.

Finally, we wish to emphasize a third point: that algorithms, however complex, are only as “correct” as the underlying assumptions and parameters we build into them. We have described our iterative algorithmic approach and we began by taking a simple visual approach. The latter helps us cognitively assess the extent of connection and disconnection, whilst the former allows us to apply statistical rigor to the process. We believe that rather than achieving a “correct solution” as Smelcer and Carmel may have argued, we have at the very least arrived at a satisficing one [23]. This represents a first step towards a better understanding of the regional economic geography of the United States and our hope is that, in due course, we can build upon these results with future data releases. In the meantime, we hope that organizations such as the US Bureau of Labor Statistics, the Regional Plan Association, the Federal Highways Administration and the US Census Bureau might find value in these methods, which take naïve delineations of megaregions and equip them with a modicum of empirical proof and precision.

## Conclusion

The results of this study point to a dynamic conclusion about the possibility of isolating coherent regional units: even within a massively-interlinked spatial structure, it is still possible to evaluate a structured geography of unitary regions. Such a regionalization is evident not only in a visual interpretation of commuter networks, but also emerges in an algorithmic partitioning of this same network. The detection of recognizable communities through this computational analysis suggests that human geography does in fact display statistically-significant patterns of structured regionalization, and that this regionalization matches interpretive descriptions of “megaregions.” Such empirical analyses provide a scaffolding on which policymakers can evaluate the appropriate territorial shape and size of districts ranging from regional transit zones to electoral constituencies. However, as shown in the interplay between the visual and algorithmic methods of assigning communities to megaregions, we must be careful not to assume that an absolute or final structure of regional divisions can be determined. Given the massive complexity of the connections inherent in national-scale commuter geography, these analyses should be understood as adumbrating broad regional patterns that must then be subject to functional and practical scrutiny.
